# Trends in Medicare payments within the first year of cervical cancer diagnosis, 2010-2019

**DOI:** 10.1093/jncics/pkaf043

**Published:** 2025-04-16

**Authors:** Mohammad A Karim, Ning Zhang, Hui Zhao, Ya-Chen Tina Shih, Lakshmi S M Kodali, Sharon H Giordano, Sanjay Shete

**Affiliations:** Department of Healthcare Administration and Policy, University of Nevada Las Vegas, Las Vegas, NV, United States; Department of Epidemiology, The University of Texas MD Anderson Cancer Center, Houston, TX, United States; Department of Health Services Research, The University of Texas MD Anderson Cancer Center, Houston, TX, United States; Department of Health Services Research, The University of Texas MD Anderson Cancer Center, Houston, TX, United States; Program in Cancer Health Economics Research, University of California Los Angeles Jonsson Comprehensive Cancer Center, Los Angeles, CA, United States; Department of Radiation Oncology, UCLA David Geffen School of Medicine, Los Angeles, CA, United States; Department of Health Policy and Management, UCLA Fielding School of Public Health, Los Angeles, CA, United States; Department of Public Health and Health Sciences, University of Michigan–Flint, Flint, MI, United States; Department of Health Services Research, The University of Texas MD Anderson Cancer Center, Houston, TX, United States; Department of Epidemiology, The University of Texas MD Anderson Cancer Center, Houston, TX, United States; Department of Biostatistics, The University of Texas MD Anderson Cancer Center, Houston, TX, United States; Division of Cancer Prevention and Population Science, The University of Texas MD Anderson Cancer Center, Houston, TX, United States

## Abstract

Assessing Medicare payment trends for cervical cancer care is important to mitigate the financial impact on Medicare. This multiyear cross-sectional study included 65 years and older cervical cancer patients in SEER registries diagnosed between 2010 and 2019 who had continuous Part A and B Medicare coverage at least 6 months before diagnosis and at least within the first year of diagnosis and were not enrolled in any Health Maintenance Organization (HMO) in this duration. The main outcomes were trends in total and service-specific mean monthly Medicare payments within the first year of a cervical cancer diagnosis. This study included 2147 cervical cancer patients. The mean Medicare payments increased from $8300 in 2010 to $8520 in 2019, largely driven by a statistically significant increase in outpatient services costs, from $1361 to $2056 (AAPC = 5.45, 95% CI = 1.38 to 9.67, *P* = .008). These findings highlight the need for policy actions to mitigate cervical-cancer-related financial impact on Medicare.

With increased adoption of cervical cancer screening, the incidence rate of invasive cervical cancer decreased by around 54% in the United States between 1973 and 2007.^1^ Unfortunately, the decrease in overall incidence rate has stagnated in recent years, with an average decrease rate of only 0.7% between 2008 and 2017.^2^ The recent number of incident cervical cancer cases is sizable, with an estimated 13 820 new cases in the United States in 2024.^2^ The current treatment landscape for cervical cancer involves a multimodal approach guided by disease stage at diagnosis, histological subtype, and patient fertility considerations. Early-stage cervical cancer is typically managed with surgery (ie, total or radical hysterectomy) or fertility-sparing procedures (ie, conization, loop electrosurgical excision procedure or trachelectomy), whereas, for locally advanced stages, concurrent chemoradiotherapy (CRT) is the standard of care.^3^^,^^4^ Additionally, immune checkpoint inhibitors have demonstrated promising results for certain subgroups of patients, which has led to the recent FDA approval of pembrolizumab plus CRT for stage III-IVA cervical cancer.^4^ Substantial costs are associated with almost all of these treatment modalities. Thus, along with the devastating health effects, cervical cancer exerts a considerable financial burden on the patients and payers in the United States.^5^^,^^6^

The cost trend for commercially insured cervical cancer patients has been examined previously,^5^ reporting an increase from 2008 to 2015.^5^ Despite covering a substantial number of cervical cancer patients, either through age or disability eligibility, the trends in Medicare payments for beneficiaries with cervical cancer have not been reported in the literature. Estimates of cost trends can inform decisionmakers of the disease burden of cervical cancer to the Medicare program and assess the potential financial impact of cost mitigation strategies. In this study, we estimated mean monthly Medicare payment trends within the first year after the diagnosis of cervical cancer for patients diagnosed between 2010 and 2019.

We used the Surveillance, Epidemiology, and End Results (SEER)-Medicare linked database with incident cases diagnosed between 2010 and 2019. SEER-Medicare is a population-based data source that linked Medicare-eligible cancer patients who resided in the SEER areas that covered around 48% of the US population.^7^^,^^8^ The follow-up time for each patient in our study was either 12 months from the month of cancer diagnosis or death, whichever was earlier. The SEER data contains patients’ demographics, tumor characteristics, and cause of death information. The Medicare data contains patients’ Medicare enrollment records and claims data for services covered, such as inpatient services, physician services, outpatient services, home health services, hospice services, and durable medical equipment.^9^

Study outcomes of interest were trends in total and service-specific mean monthly Medicare payments within the first year of a cervical cancer diagnosis. The service-specific Medicare payments were obtained for: (i) inpatient/skilled nursing facility (SNF) services, which included services received in an inpatient setting or in a SNF; (ii) physician/supplier services, which included services received from physicians and other noninstitutional providers; (iii) outpatient services, which included services received from institutional outpatient providers; (iv) durable medical equipment (DME), which included utilization of durable medical equipment and oral equivalents of IV chemotherapies; (v) home health services, which included services received from home health agencies; and (vi) hospice services, which included services received from hospice providers.^9^

Cervical cancer was identified using the International Classification of Diseases for Oncology, Third Edition (ICD-O–3) codes C53.0–53.9.^6^ All cancer patients who were 65 years or older at the time of diagnosis, had cervical cancer as the first and only primary cancer diagnosed between 2010 and 2019, had continuous Part A and B Medicare coverage from 6 months before diagnosis to at least the first year of diagnosis or death (whichever was earlier) but had no HMO coverage during this period were included in the study. We excluded cervical cancer diagnosis confirmed solely from autopsy or death certificate because follow-up care information was not available for those cases.^10^

We estimated adjusted mean monthly total and service-specific Medicare payments using a generalized linear model (GLM) with log link and gamma distribution or a 2-part regression model, with the first part as a logistic regression and the second part as a GLM model with log link and gamma distribution.^11^^,^^12^ A 2-part model is considered an appropriate method to model a dependent variable with a large number of zero values (such as certain health-care claims), and, hence, was used in our analyses for the services with about 10% or more zero dollar claims (ie, inpatient/SNF, outpatient, DME, home health and hospice services).^11^ Age at diagnosis, race/ethnicity, marital status, census tract educational attainment indicator, census tract poverty indicator, urban/rural status, year of diagnosis, National Cancer Institute (NCI) comorbidity index (calculated using 6 months of prediagnosis claims), cancer stage, and survival status in the first year after diagnosis were used as covariates in our analyses. Census tract educational attainment indicator and census tract poverty indicator were defined using socioeconomic status (SES) variables provided in the Census Tract File from SEER-Medicare. In the census tract educational attainment indicator variable, Quartile 1 refers to the quartile of tracts with the lowest percentage, whereas Quartile 4 refers to the quartile of tracts with the highest percentage of 25 years or older residents without a high school diploma. Similarly, in the census tract poverty indicator variable, Quartile 1 refers to the lowest quartile of residents living below the poverty line, whereas Quartile 4 refers to the highest quartile of tracts with residents below the poverty line.

Trend analysis was conducted using the Joinpoint Trend Analysis software available from the NCI.^13^ Adjusted mean values estimated from the regression models for each service type were used as the input values in the Joinpoint analyses. The measure annual percent change (APC) indicated an increasing or decreasing trend over time, and the measure average annual percentage change (AAPC) provided a summary for multiple APC measures over a fixed time period.^14^ Medicare payments were inflation-adjusted to 2023 using Consumer Price Indices for medical care.^15^^,^^16^ We set statistical significance as a 2-sided *P* < .05. All analyses were performed using SAS 9.4 (SAS Institute, Inc., Cary, NC) and Stata 15 (StataCorp, College Station, TX) software. The study was approved by MD Anderson Cancer Center Institutional Review Board (2020-1249).

A total of 2147 cervical cancer patients were included in our study with a mean age of 75.24 years (SD, 7.61 years). Most of the patients were non-Hispanic White (77.18%, *n* = 1657), and most (59.39%, *n* = 1275) lived in metro areas with a population greater than 1 million. A total of 32.51% (*n* = 698) of the patients died within a year of a cancer diagnosis. Table 1 represents sociodemographic and clinical characteristics in detail.

**Table 1.** Characteristics of cervical cancer patients with Medicare coverage diagnosed between 2010 and 2019 - Surveillance, Epidemiology, and End Results–Medicare database (*n *= 2147)

Variable	*n*	%
Age at diagnosis		
65 to <70 years	611	28.46
70 to <75 years	540	25.15
75 to <80 years	393	18.3
≥80 years	603	28.09
Race/ethnicity		
Non-Hispanic White	1657	77.18
Non-Hispanic Black	239	11.13
Hispanic	140	6.52
Asian	**	**
Other	**	**
Marital status		
Not married	753	35.07
Married	493	22.96
Unknown	901	41.97
Urban/rural status		
Metro >1 million	1275	59.39
Metro 250 000 to 1 million	376	17.51
Metro <250 000	169	7.87
Non-Metro	327	15.23
Census tract educational attainment indicator		
Quartile 1	593	27.62
Quartile 2	552	25.71
Quartile 3	506	23.57
Quartile 4	496	23.1
Census tract poverty indicator		
Quartile 1	579	26.97
Quartile 2	547	25.48
Quartile 3	525	24.45
Quartile 4	496	23.1
Year of diagnosis		
2010	193	8.99
2011	236	10.99
2012	213	9.92
2013	214	9.97
2014	240	11.18
2015	222	10.34
2016	205	9.55
2017	225	10.48
2018	203	9.46
2019	196	9.13
NCI comorbidity index		
0	1252	58.31
>0 to 2	655	30.51
>2 to 4	179	8.34
>4	61	2.84
Cancer stage		
In situ/Localized	537	25.01
Regional	879	40.94
Distant	556	25.9
Unstaged/Unknown	175	8.15
Died within 1 year of diagnosis	698	32.51

** The frequency and percentage for the “Asian” and “Other” race/ethnicity categories are suppressed in accordance with the SEER-Medicare requirement to suppress cells with a number of observations less than 11.

In the census tract educational attainment indicator variable, Quartile 1 refers to the quartile of tracts with the lowest percentage of 25 years or older residents without a high school diploma, whereas Quartile 4 refers to the quartile of tracts with the highest percentage of 25 years or older residents without a high school diploma. In the census tract poverty indicator variable, Quartile 1 refers to the lowest quartile of tracts with residents living below the poverty line, whereas Quartile 4 refers to the highest quartile of tracts with residents living below the poverty line.

For cervical cancer patients diagnosed between 2010 and 2019, the mean monthly total Medicare payments increased from $8300 to $8520, with a peak amount of $9237 in 2017 (Table S1). Importantly, the increasing trend in total Medicare payments was driven primarily by a statistically significant increasing trend in outpatient services over the study period (from $1361 to $2056; AAPC = 5.45, 95% CI = 1.38 to 9.67, *P* = .008), with a steeper increase between 2010 and 2015 (from $1361 to $1803; APC = 7.11, 95% CI = 0.86 to 13.76, *P* = .03; Figure 1 and Table S2). Inpatient/SNF services consistently incurred the highest amounts, whereas DME services consistently incurred the lowest amounts of Medicare payments (Figure S1).

**Figure 1.**
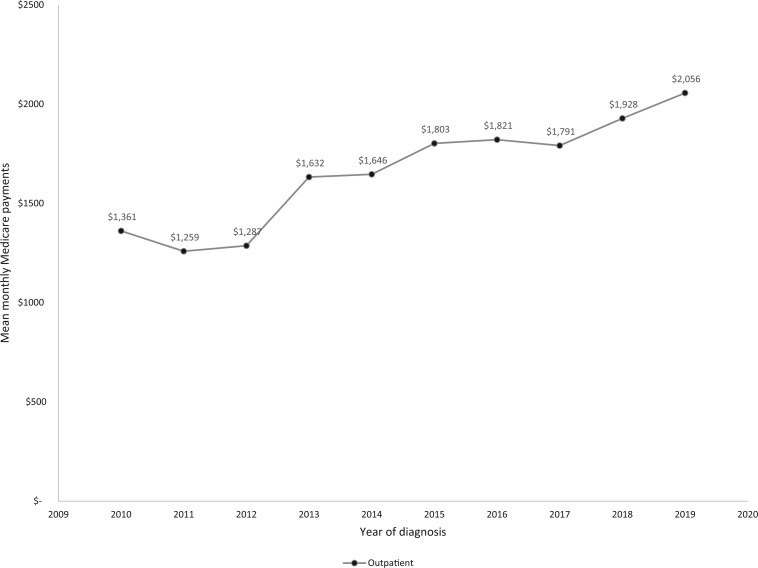
Mean monthly Medicare payments for outpatient services in the year after diagnosis for cervical cancer patients with Medicare coverage, diagnosed between 2010 and 2019 - Surveillance, Epidemiology, and End Results–Medicare database (*n* = 2147). ^a^Outpatient services included services received from institutional outpatient providers. ^b^Mean monthly Medicare payments were adjusted for age at diagnosis, race/ethnicity, marital status, census tract level educational attainment, census tract level poverty, urban/rural status, year of diagnosis, National Cancer Institute comorbidity index (calculated using 6 months prediagnosis claims), cancer stage, and survival status in the first year after diagnosis. ^c^All reported dollar values are inflation-adjusted to the 2023 US Dollar.

In this retrospective analysis of the SEER-Medicare database, we found an increasing trend in Medicare payments for outpatient services for cervical cancer patients in recent years. This increase is in line with the increasing trajectory of national Medicare spending, which almost doubled between 2008 and 2022, and overall outpatient spending for Medicare enrollees, which saw a consistent 5.5% average increase per year between 2013 and 2023.^17^

Reports of cervical cancer costs and cost trends are sparse in the literature. In an analysis of the Medical Expenditure Panel Survey, Shah et al. estimated total costs for cervical cancer using pooled 2006-2016 data; however, no trends were reported.^18^ Their estimated per person per year total costs and outpatient costs of $10 031 and $1610, respectively, were substantially lower than the respective Medicare payment amounts estimated in our study.^18^ A younger study population, differences in insurance types covered, and a different referent year for inflation adjustment (ie, 2017) may have contributed to these differences. Another study examining cervical cancer survivors in Texas reported $9827 annual total Medicare payments, which is also substantially lower than our estimates.^19^ Potential reasons for this difference include a different set of services examined (only inpatient, outpatient, and hospice care) and an earlier referent year for inflation adjustment (ie, 2009). For commercially insured cervical patients in Texas, Lairson et al. estimated outpatient costs of $39 577 per annum in the year after diagnosis using pooled 2011-2014 MarketScan data.^20^ This estimated amount, which was based on payments to providers before applying any deductibles or copayment, is higher than outpatient costs estimated in our study, which may reflect higher reimbursement in commercial insurances than Medicare.

One of the few studies reporting changes in cervical cancer costs over time was the Branco et al. study, which investigated commercially insured cervical cancer patients and reported an increase in costs in the year 2015 compared with 2008 for patients receiving either surgery or radiation.^5^ Our results show increasing trends for outpatient services in Medicare between the years 2010 and 2019, with a steep increase between 2010 and 2015. The 2010-2015 time period overlaps with the early adoption period of robotic gynecologic surgeries in the United States;^21^ however, our study was not designed to identify the impact of specific treatment modalities on costs. Future studies should investigate whether the increasing trend in Medicare payments for outpatient services to cervical cancer patients was driven by system-level factors or changes in treatment patterns.

Our study has a few limitations. We did not examine prescription medication payments because excluding patients without continuous Medicare Part D coverage would substantially reduce the sample size. Additionally, our study is generalizable only to fee-for-service Medicare beneficiaries with cervical cancer residing in the SEER regions, not to the general US population. Although a substantial portion of Medicare enrollees are enrolled in the HMO plans (ie, Medicare Advantage), the SEER-Medicare database does not include any cost-related data for those enrollees. This is because the source of the claims portion of SEER-Medicare data, the Centers for Medicare and Medicaid Services, does not release the cost-related data. Due to this inherent limitation of the SEER-Medicare database, we could not examine Medicare HMO enrollees in our study. Moreover, our study did not identify cancer-attributable expenses but rather examined overall Medicare payments. Nonetheless, multiyear SEER-Medicare offered a unique database that provided population-based information for cancer patients with Medicare coverage, a notable strength of our study.

In conclusion, our finding of the upward trend in Medicare payments for outpatient services presents a point of concern. Rising per capita costs and increasing number of patients may fuel the future increase in the total financial burden of cancers, straining the Medicare system. Therefore, focused policy actions are warranted to mitigate the financial impact of cervical cancer care on Medicare.

## Supplementary Material

pkaf043_Supplementary_Data

## Data Availability

The SEER-Medicare data used in this study were obtained from the National Cancer Institute (NCI) and are not publicly available. Researchers are required to complete an application process with the NCI using a prescribed format to request the data.
