# Supplementary material for: Trends in Medicare payments within the first year of cervical cancer diagnosis, 2010-2019
Source: JNCI Cancer Spectr. 2025 Apr 16;9(3):pkaf043. doi: 10.1093/jncics/pkaf043 (PMC12097483; doi:10.1093/jncics/pkaf043)
Supplement: pkaf043_Supplementary_Data [file pkaf043_supplementary_data.docx]

Supplementary Figure 1 – Mean monthly total Medicare payments (Panel A), and Medicare payments for inpatient/SNF services (Panel B), physician services (Panel C), hospice services (Panel D), home health services (Panel E) and DME services (Panel F); Inflation adjusted to 2023 US Dollar; Adjusted for age at diagnosis, race/ethnicity, marital status, census tract, educational attainment indicator, census tract poverty indicator, urban/rural status, year of diagnosis, National Cancer Institute (NCI) comorbidity index, cancer stage, and survival status in the first year after diagnosis.

| Panel A | Panel B |
| --- | --- |

| Panel C | Panel D |
| --- | --- |
|  |  |
| Panel E | Panel F |
|  |  |

Supplementary Figure 1. Mean monthly total Medicare payments (Panel A), and Medicare payments for inpatient/SNF services (Panel B), physician/supplier services (Panel C), home health services (Panel E), hospice services (Panel D) and durable medical equipment (DME) services (Panel F), in the year after diagnosis for cervical cancer patients with Medicare coverage, diagnosed between 2010 and 2019 - Surveillance, Epidemiology, and End Results–Medicare database (N=2147)

Footnotes: ^1^ Inpatient/skilled nursing facility (SNF) services included services received in an inpatient setting or in a SNF; physician/supplier services included services received from physicians and other non-institutional providers; durable medical equipment (DME) services included utilization of durable medical equipment and oral equivalents of IV chemotherapies; home health services included services received from home health agencies; and, hospice services included services received from hospice providers

^2^ Mean monthly Medicare payments were adjusted for age at diagnosis, race/ethnicity, marital status, census tract level educational attainment, census tract level poverty, urban/rural status, year of diagnosis, National Cancer Institute comorbidity index (calculated using 6 months pre-diagnosis claims), cancer stage and survival status in the first year after diagnosis.

^3^ The total dollar values may not equal to the sum of the components because the adjusted totals estimated from separate regression models are presented in the figure.

**Supplementary Table 1.** Mean monthly total Medicare payments and Medicare payments for outpatient services, inpatient/SNF services, physician services, hospice services, home health services, and DME services in the period between 2010-2019; Inflation adjusted to 2023 US Dollar; Adjusted for age at diagnosis, race/ethnicity, marital status, census tract, educational attainment indicator, census tract poverty indicator, urban/rural status, year of diagnosis, NCI comorbidity index, cancer stage, and survival status in the first year after diagnosis.

|  | **Medicare payments by services type, 2023 US$ (95% CI)** | | | | | | |
| --- | --- | --- | --- | --- | --- | --- | --- |
| **Year of diagnosis** | **Total** | **Outpatient** | **Inpatient/SNF** | **Physician** | **Hospice** | **Home health** | **DME** |
| 2010 | 8,300 (7,366-9,235) | 1,361 (1,171-1,552) | 3,985 (3,135-4,836) | 2,123 (1,844-2,401) | 659 (419-899) | 198 (137-258) | 59 (25-93) |
| 2011 | 7,939 (7,127-8,751) | 1,259 (1,099-1,419) | 4,336 (3,531-5,142) | 1,903 (1,676-2,129) | 398 (239-558) | 162 (117-207) | 46 (22-70) |
| 2012 | 9,055 (8,089-10,022) | 1,287 (1,115-1,459) | 4,941 (4,011-5,870) | 2,059 (1,802-2,316) | 587 (374-800) | 229 (171-287) | 34 (17-51) |
| 2013 | 7,997 (7,143-8,851) | 1,632 (1,412-1,853) | 3,523 (2,808-4,238) | 1,767 (1,547-1,988) | 905 (636-1,174) | 194 (136-252) | 17 (6-28) |
| 2014 | 8,348 (7,499-9,197) | 1,646 (1,439-1,854) | 4,229 (3,472-4,986) | 1,763 (1,555-1,971) | 436 (294-577) | 251 (193-309) | 24 (12-35) |
| 2015 | 8,931 (7,989-9,873) | 1,803 (1,569-2,036) | 4,418 (3,504-5,333) | 1,812 (1,589-2,035) | 721 (486-956) | 177 (119-234) | 16 (7-24) |
| 2016 | 9,144 (8,136-10,152) | 1,821 (1,564-2,078) | 4,297 (3,434-5,160) | 1,912 (1,667-2,158) | 743 (506-980) | 189 (134-244) | 18 (7-29) |
| 2017 | 9,237 (8,273-10,201) | 1,791 (1,559-2,023) | 4,413 (3,574-5,251) | 2,113 (1,856-2,370) | 590 (399-780) | 184 (134-235) | 13 (6-20) |
| 2018 | 8,203 (7,294-9,112) | 1,928 (1,665-2,192) | 3,321 (2,603-4,038) | 1,969 (1,714-2,224) | 465 (290-640) | 131 (87-176) | 16 (6-26) |
| 2019 | 8,520 (7,561-9,479) | 2,056 (1,780-2,332) | 3,832 (2,972-4,692) | 1,831 (1,591-2,071) | 504 (316-691) | 193 (135-251) | 18 (7-29) |

**Supplementary Table 2.** Annual Percent Change (APC) in mean monthly total Medicare payments and Medicare payments for outpatient services, inpatient/SNF services, physician services, hospice services, home health services, and DME services with one Joinpoint estimated between 2010-2019. Mean monthly total Medicare payments and Medicare payments for each service type (inflation-adjusted to 2023 US dollar), adjusted for age at diagnosis, race/ethnicity, marital status, census tract, educational attainment indicator, census tract poverty indicator, urban/rural status, year of diagnosis, NCI comorbidity index, cancer stage, and survival status in the first year after diagnosis estimated from the regression analyses were the input in the Joinpoint analyses.

|  | **Segment** | **Lower Endpoint (year)** | **Upper Endpoint (year)** | **APC (95% CI)** | **P-value** |
| --- | --- | --- | --- | --- | --- |
| **Total** | 1 | 2010 | 2017 | 1.57 (-1.06 to 4.28) | 0.19 |
|  | 2 | 2017 | 2019 | -4.31 (-21.84 to 17.15) | 0.60 |
| **Outpatient** | 1 | 2010 | 2015 | 7.11 (0.86 to 13.76) | 0.03 |
|  | 2 | 2015 | 2019 | 3.40 (-5.34 to 12.94) | 0.38 |
| **Inpatient/SNF** | 1 | 2010 | 2017 | -0.06 (-5.50 to 5.69) | 0.98 |
|  | 2 | 2017 | 2019 | -8.60 (-42.17 to 44.45) | 0.63 |
| **Physician** | 1 | 2010 | 2013 | -4.89 (-16.40 to 8.20) | 0.36 |
|  | 2 | 2013 | 2019 | 1.47 (-2.75 to 5.87) | 0.42 |
| **Hospice** | 1 | 2010 | 2016 | 3.42 (-15.23 to 26.18) | 0.68 |
|  | 2 | 2016 | 2019 | -12.72 (-51.40 to 56.77) | 0.58 |
| **Home health** | 1 | 2010 | 2014 | 5.28 (-13.71 to 28.46) | 0.54 |
|  | 2 | 2014 | 2019 | -6.62 (-19.44 to 8.24) | 0.29 |
| **DME** | 1 | 2010 | 2013 | -31.71 (-51.29 to -12.47) | 0.005 |
|  | 2 | 2013 | 2019 | -5.10 (-16.67 to 33.14) | 0.70 |
